# Influence of baseline neurologic severity on disease progression and the associated disease-modifying effects of tafamidis in patients with transthyretin amyloid polyneuropathy

**DOI:** 10.1186/s13023-018-0947-7

**Published:** 2018-12-17

**Authors:** Leslie Amass, Huihua Li, Balarama K. Gundapaneni, Jeffrey H. Schwartz, Denis J. Keohane

**Affiliations:** 1 Pfizer Inc., 500 Arcola Road, Collegeville, PA 19426 USA; 2 Pfizer Inc., 235 E 42nd St, New York, NY 10017 USA; 3 InVentiv Health Inc., 1 Van de Graaff Drive, Burlington, MA 01803 USA; 4 Pfizer Inc., 558 Eastern Point Rd, Groton, CT 06340 USA

**Keywords:** Transthyretin, Amyloidosis, Baseline severity, Disease progression, Polyneuropathy, NIS-LL, Val30Met, ATTR

## Abstract

**Background:**

Emerging evidence suggests that several factors can impact disease progression in transthyretin amyloid polyneuropathy (ATTR-PN). The present analysis used longitudinal data from Val30Met patients participating in the tafamidis (selective TTR stabilizer) clinical development program to evaluate the impact of baseline neurologic severity on disease progression in ATTR-PN.

**Methods:**

A linear mixed-effects model for repeated measures (MMRM) was constructed using tafamidis and placebo data from the intent-to-treat Val30Met population of the original registration study as well as tafamidis data from the two consecutive open-label extension studies. The second extension study is ongoing, but a prospectively-planned interim analysis involving a cleaned and locked database was conducted (cut-off: December 31, 2014). Val30Met patients are presented by treatment groups as those who received tafamidis during the registration and open-label studies (T-T group), or who received placebo during the registration study and were switched to tafamidis in the open-label studies (P-T group). Neurologic functioning was assessed at baseline and subsequent visits using the Neuropathy Impairment Score–Lower Limbs (NIS-LL). The analysis focused on the disease trajectory over the first 18 months of treatment.

**Results:**

The T-T (*n* = 64) and P-T (*n* = 61) cohorts were predominantly Caucasian and presented with early-stage neurologic disease (mean [standard deviation] baseline NIS-LL values were 8.4 [11.4] and 11.4 [13.5], respectively). The MMRM analysis demonstrated that baseline severity is an independent significant predictor of disease progression in addition to the treatment effect: patients with a lower baseline NIS-LL showed less progression than those with a higher baseline NIS-LL (*p* < 0.0001). Neurologic progression in the T-T group was less than in the P-T group across all levels of baseline NIS-LL (*p* = 0.0088), and the degree of separation increased over the 18-month period. Similar results were seen with the NIS-LL muscle weakness subscale.

**Conclusions:**

This analysis of patients with Val30Met ATTR-PN demonstrates that neurologic disease progression strongly depends on baseline neurologic severity and illustrates the disease-modifying effect of tafamidis relative to placebo across a range of baseline levels of neurologic severity and treatment durations. These data also underscore the benefit of early diagnosis and treatment with tafamidis in delaying disease progression in ATTR-PN.

**Trial Registration:**

NCT00409175, NCT00791492 and NCT00925002 registered 08 December 2006, 14 November 2008 (retrospectively registered), and 19 June 2009, respectively.

**Electronic supplementary material:**

The online version of this article (10.1186/s13023-018-0947-7) contains supplementary material, which is available to authorized users.

## Background

Transthyretin amyloid polyneuropathy (ATTR-PN) is a rare, systemic condition characterized by *TTR* gene mutations that result in pervasive amyloid accumulation in peripheral nerve tissue and vital organs [1]. The illness carries a high symptom burden, manifesting as diverse and progressively debilitating neurologic and autonomic symptoms, frequently with cardiac involvement, and a severely shortened life span [1, 2]. A current review of the prevalence of ATTR-PN suggests the global prevalence to be substantially higher than current widely cited estimates of 5000–10,000 persons and perhaps as high as ~ 39,000 persons worldwide, pointing to a significantly higher prevalence of this disease than previously thought [3].

Over 100 genotypes have been identified, with considerable phenotypic heterogeneity both within and across variants [4, 5] that can make diagnosing the disease and monitoring its progression challenging. Clinicians and researchers need optimal ways to define disease progression and more sensitively assess a patient’s response to treatment. Emerging evidence suggests that neurologic progression in ATTR-PN is not fixed, and factors such as baseline neurologic impairment, age of onset, and genotype are critical to assessing the impact of treatment [6–9].

The objective of this analysis was to construct a model for assessing neurologic progression in ATTR-PN. Longitudinal data from Val30Met patients participating in the tafamidis clinical development program [10–12] were used to better understand the relationship between baseline disease burden and neurologic progression in ATTR-PN. Tafamidis, a highly selective TTR stabilizer, is approved to delay neurologic progression in adult patients with ATTR-PN with current market authorizations in several countries across Europe, Latin America, and Asia [13].

## Methods

### Analysis design and patient data

A statistical model was constructed using tafamidis and placebo data from Val30Met patients in the intent-to-treat (ITT) population of the 18-month, double-blind, registration study (ClinicalTrials.gov identifier: NCT00409175) [10], as well as tafamidis data obtained from the same Val30Met patients who subsequently enrolled in the two consecutive open-label extension studies (NCT00791492 and NCT00925002) [11, 12]. The first extension study comprised patients who completed the registration study and were eligible to receive tafamidis in a 12-month, open-label extension study. Upon completion of this 12-month study, patients were then eligible to enter an ongoing long-term, open-label study of tafamidis. A formal, prospectively planned, interim analysis (cut-off date December 31, 2014) that included a cleaned and locked database of all safety and efficacy variables was conducted [12]. Inclusion criteria for the ITT population in the registration study have been reported previously and included all patients who received at least one dose of once-daily oral study medication (placebo or tafamidis meglumine 20 mg) and who had at least one post baseline efficacy assessment for both the Neuropathy Impairment Score – Lower Limbs (NIS-LL) and the Norfolk Quality of Life – Diabetic Neuropathy questionnaire, or who discontinued the study due to death or liver transplant [10]. Details on study design, methodology, and study participants are available in their respective primary publications [10–12]. All studies were conducted with the approval of local institutional review boards or independent ethics committees (Additional file 1: Table S1), and in accordance with the Declaration of Helsinki, the International Conference on Harmonisation Guideline for Good Clinical Practice and local regulatory requirements. All patients provided written informed consent.

Val30Met patients are presented by treatment group as those who received tafamidis during both the registration study and open-label studies (tafamidis-to-tafamidis [T-T] group) and those who received placebo during the registration study and were switched to tafamidis on entry into the first open-label extension study (placebo-to-tafamidis [P-T] group).

### Outcome measure used for predictive modeling: NIS-LL

Across studies, the neurologic functioning of patients was assessed at baseline (the first study visit of the 18-month, double-blind, registration study) and subsequent visits using the NIS-LL (scale ranges from 0 [normal functioning] to 88 [total impairment]), a sensitive and valid measure of neurologic functioning in the lower limbs (which are commonly affected in the early stages of ATTR-PN, especially in a Val30Met patient population) [14]. The NIS-LL muscle weakness subscale, which was shown in these studies to be the primary contributor to changes in the overall NIS-LL, was also assessed (scale ranges from 0 to 64) [12]. The muscle weakness subscale includes an assessment of hip flexion, hip extension, knee flexion, knee extension, ankle dorsiflexors, ankle planter flexors, toe extensors, and toe flexors, each of which is scored on a scale from 0 (normal) to 4 (paralysis) [10].

### Statistical analysis

Slope analyses of NIS-LL and NIS-LL muscle weakness were performed separately using a linear mixed-effects model for repeated measures (MMRM), which adjusts for the effect of baseline covariate and tests for differences in disease progression time-slope between groups defined by treatment and phase (four groups defined as P-T in first 18 months of double-blind study, P-T in open-label extension studies, T-T in first 18 months of double-blind study, and T-T in open-label extension studies). The MMRM was used as it analyzes data from all participants (rather than completers) in the estimation of the slope parameter by contributing data at the time points where observations were collected. The model was constructed with treatment and baseline effects, and the two-way interactions of each of these variables with time, as fixed effects; the slope and intercept for each patient were random effects. Time was defined as the number of months from the first dose in the registration study until the day of assessment. An unstructured covariance matrix was used to model the independence of the slope and intercept parameters. Parameters were estimated using restricted maximum likelihood. The primary tests of interest were the significance of the independent effects of baseline and treatment on disease progression time-slope via testing on baseline-by-time interaction and treatment-by-time interaction.

Although the statistical model was based on aggregate data from 5.5 years of exposure across the three clinical trials described above, the current report focused on the first 18 months of treatment when patients received either placebo or tafamidis.

## Results

### Patient characteristics

The baseline demographic and clinical characteristics of the T-T (*n* = 64) and P-T (*n* = 61) groups have been described previously [10]. Both treatment groups contained an approximately equal number of men and women (% male: T-T, 50%; P-T, 43%) of similar age (mean ± standard deviation [SD], years: T-T, 39.8 ± 12.7; P-T, 38.4 ± 12.9) and modified body mass index (mean ± SD, [g/L] x [kg/m^2^]: T-T, 1004.6 ± 165.2; P-T, 1011.5 ± 212.9), were predominantly Caucasian (T-T, 88%; P-T, 89%) and presented with early-stage neurologic disease at baseline (mean NIS-LL ± SD: T-T, 8.4 ± 11.4; P-T, 11.4 ± 13.5; mean symptom duration ± SD, months: T-T, 47.0 ± 48.4; P-T, 34.7 ± 32.9) [10].

### Disease progression in relation to baseline neurologic severity and the effect of treatment

The MMRM analysis demonstrates that baseline severity is an independent significant predictor of disease progression in addition to the treatment effect. The results are presented in Table 1, where the baseline coefficient representing the mean change (standard error, SE) in slope associated with an increase of one point in baseline is 0.0096 (0.0016) per month for NIS-LL (*p* < 0.0001) and 0.0119 (0.0019) per month for muscle weakness (*p* < 0.0001). This means that a one-point increase in baseline NIS-LL is associated with a faster mean NIS-LL increase (clinical decline) of 0.0096 (0.0016) per month. Likewise, a one-point increase in baseline in NIS-LL muscle weakness is associated with a faster mean muscle weakness increase (clinical decline) of 0.0119 (0.0019) per month.

**Table 1:** Effect of baseline severity and treatment on NIS-LL and NIS-LL muscle weakness slopes per month

	NIS-LL			NIS-LL muscle weakness		
Effect	Coefficient	Standard error	*p*-value	Coefficient	Standard error	*p*-value
Baseline^a^	0.0096	0.0016	< 0.0001	0.0119	0.0019	< 0.0001
Treatment group^b^						
P-T during double-blind phase	0.3249	0.0453	0.0088^c^	0.1905	0.0360	0.0132^c^
T-T during double-blind phase	0.1558	0.0448		0.0636	0.0356	

^a^Represents the mean change in the slope per month associated with an increase in the respective baseline covariate equal to one unit

^b^Represents baseline-adjusted slope per month corresponding to the treatment group. Values for the open label phase of the extension study are not shown

^c^*p*-value for slope comparison between tafamidis and placebo during the 18-month double-blind phase of the registration study

*NIS-LL* Neuropathy Impairment Score − Lower Limbs; *P-T* placebo-to-tafamidis; *T-T* tafamidis-to-tafamidis

**Fig. 1:**
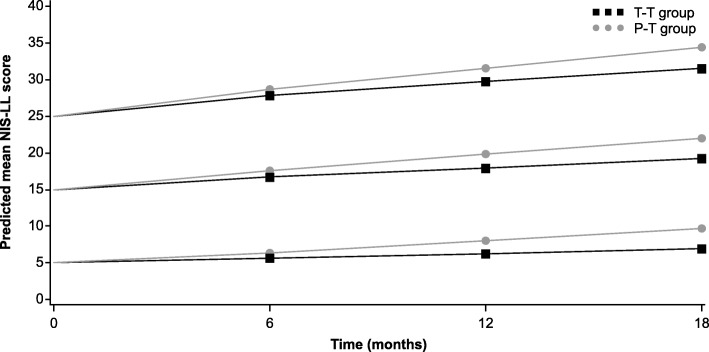
NIS-LL progression in relation to baseline severity and the effect of treatment. Baseline NIS-LL scores of 5, 15, and 25 were chosen for illustration and were used as the zero time point. Values for Months 6, 12, and 18 were estimated using the linear mixed-effects model. *NIS-LL* Neuropathy Impairment Score − Lower Limbs; *P-T* placebo-to-tafamidis; *T-T* tafamidis-to-tafamidis

**Fig. 2:**
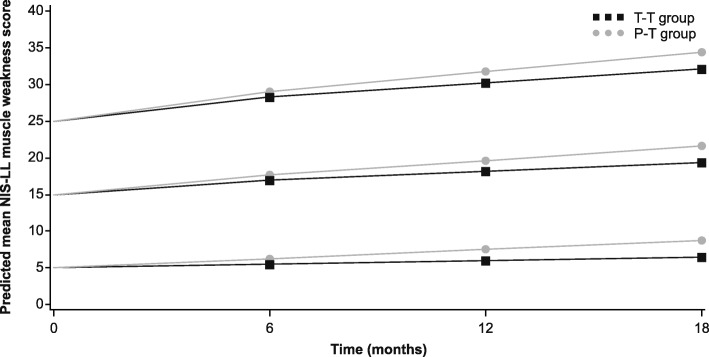
NIS-LL muscle weakness progression in relation to baseline severity and the effect of treatment. Baseline NIS-LL muscle weakness scores of 5, 15, and 25 were chosen for illustration and were used as the zero time point. Values for Months 6, 12, and 18 were estimated using the linear mixed-effects model. *NIS-LL* Neuropathy Impairment Score − Lower Limbs; *P-T* placebo-to-tafamidis; *T-T* tafamidis-to-tafamidis

Disease progression in relation to baseline neurologic severity and the effect of treatment is depicted in Figs. 1 (NIS-LL) and 2 (NIS-LL muscle weakness). Baseline scores of 5, 15, and 25 were chosen for illustration, and the data from the first 18 months were used only to illustrate the baseline effect on slope of NIS-LL and NIS-LL muscle weakness in the tafamidis versus placebo groups (as shown in Figs. 1 and 2 and Tables 2 and 3). After 18 months, the placebo patients from the registration trial were switched to active treatment and followed in the extension study with tafamidis. Thus, the treatment comparison of tafamidis with placebo was only performed up to Month 18.

**Table 2:** Estimated NIS-LL at Months 6, 12, and 18, according to baseline neurologic severity and treatment group

Baseline NIS-LL	Treatment group	Month 6	Month 12	Month 18
5	T-T	5.6 (0.5)	6.3 (0.6)	6.9 (0.7)
	P-T	6.4 (0.5)	8.1 (0.6)	9.8 (0.8)
15	T-T	16.7 (0.5)	18.0 (0.6)	19.2 (0.8)
	P-T	17.6 (0.5)	19.8 (0.6)	22.0 (0.7)
25	T-T	27.9 (0.6)	29.7 (0.7)	31.5 (0.9)
	P-T	28.7 (0.6)	31.5 (0.7)	34.3 (0.9)

Data are shown as mean (standard error)

*NIS-LL* Neuropathy Impairment Score − Lower Limbs; *P-T* placebo-to-tafamidis; *T-T* tafamidis-to-tafamidis

**Table 3:** Estimated NIS-LL muscle weakness at Months 6, 12, and 18, according to severity of baseline muscle weakness and treatment group

Baseline NIS-LL muscle weakness	Treatment group	Month 6	Month 12	Month 18
5	T-T	5.5 (0.4)	6.0 (0.4)	6.5 (0.6)
	P-T	6.3 (0.4)	7.5 (0.4)	8.8 (0.6)
15	T-T	16.9 (0.5)	18.1 (0.6)	19.3 (0.7)
	P-T	17.7 (0.5)	19.6 (0.6)	21.6 (0.7)
25	T-T	28.3 (0.8)	30.2 (0.8)	32.2 (1.0)
	P-T	29.1 (0.7)	31.7 (0.8)	34.4 (1.0)

Data are shown as mean (standard error). The reason for the slightly higher mean scores in muscle weakness at certain time points (compared with the overall NIS-LL score) can be explained by the fact that separate models were conducted on NIS-LL and NIS-LL muscle weakness, which makes cross-comparisons problematic. In addition, comparable baseline scores on the two endpoints can be very different in terms of reflecting disease severity and progression; a patient with a muscle weakness score of 15 would likely be more severely ill and progress more rapidly on this scale than a patient with a comparable baseline of 15 on the total NIS-LL (this was confirmed by the present analysis where the baseline coefficient for NIS-LL muscle weakness was greater than the coefficient for NIS-LL, suggesting faster clinical decline in muscle weakness than NIS-LL within the same period, as baseline increases)

*NIS-LL* Neuropathy Impairment Score − Lower Limbs; *P-T* placebo-to-tafamidis; *T-T* tafamidis-to-tafamidis

Although the slope changes appear small relative to the differences in baseline scores (Figs. 1 and 2), the baseline coefficient from the model for NIS-LL translates to an increase of 1.2 and 2.3 points (clinical decline) in NIS-LL per year faster in patients with a baseline of 15 and 25 points, respectively, compared with patients with a baseline of 5 points. Similarly, for NIS-LL muscle weakness, the mean score increases (clinical decline) by 1.4 and 2.9 points per year faster in patients with baseline muscle weakness of 15 and 25 points, respectively, compared with patients with baseline muscle weakness of 5 points. These changes in slope can be further contextualized by deriving the predicted change from baseline to Month 18 from the values in Tables 2 and 3 (value at Month 18 minus value at baseline). As the baseline scores increase, the clinical decline in NIS-LL and NIS-LL muscle weakness becomes greater, and in some cases almost doubles; for example, when comparing the predicted change for NIS-LL of patients in the P-T group with a baseline score of 5 versus 25, the estimate increases (clinical decline) from 4.8 points to 9.3 points, respectively (Table 2).

Across all levels of baseline neurologic severity, the rate of disease progression as measured by NIS-LL in the T-T group was significantly less than the rate in the P-T group (*p* = 0.0088). On average, the estimated NIS-LL was lower in the T-T group than in the P-T group, and the separation between them increased over time during the 18 months by an extent consistent with the time course of the disease-modifying effects of tafamidis. Similar results were observed for NIS-LL muscle weakness (Fig. 2, Table 3). Across all levels of baseline muscle weakness severity, the rate of disease progression in the T-T group was significantly less than the rate in the P-T group (*p* = 0.0132). On average, the estimated NIS-LL muscle weakness score was lower in the T-T group than in the P-T group, and the separation between them increased over time during the 18 months.

## Discussion

A linear mixed-effects model for repeated measures was applied to longitudinal neurologic data from Val30Met ATTR-PN patients participating in the tafamidis clinical development program to better elucidate the impact of baseline neurologic severity on disease progression in ATTR-PN. The analysis illustrates the disease-modifying effect of tafamidis treatment relative to placebo (T-T versus P-T groups) across a range of baseline levels of neurologic severity and treatment durations. The differences in the slope (rate of change) of disease progression between T-T and P-T groups support an increasing clinical benefit from tafamidis treatment over time. The relationship between increasing baseline disease severity and disease progression was observed in patients across treatment groups over a 5.5-year period. Overall, the critical role of baseline disease severity on disease progression in ATTR-PN was shown, and the value of tafamidis for treating Val30Met patients with ATTR-PN further confirmed.

The results are generally consistent with findings from other tafamidis clinical studies. A recent post-hoc analysis of a homogeneous cohort of Val30Met patients with mild neurologic impairment at treatment start (NIS-LL ≤ 10), from the same source studies used in the present analysis, illustrated the benefits of early identification and treatment with tafamidis for delaying neurologic progression for up to 5.5 years [9]. The beneficial effects of tafamidis on neurologic progression (as measured by changes in NIS-LL) over periods of at least 1 year have also been reported in Val30Met patients with late-onset disease [15].

The present analysis was undertaken to better elucidate the impact of baseline severity on disease trajectory in ATTR-PN. The results broaden our understanding of the relevance of baseline disease burden on neurologic progression in ATTR-PN. Such information may help clinicians better assess the impact of disease-modifying medicines in their patients. Likewise, understanding the importance of baseline disease severity in disease progression can inform clinical trial methodology and improve the interpretation of treatment effects in ATTR-PN.

### Limitations

The results and interpretation of this analysis carry the inherent limitations associated with a post-hoc analysis and the combining of data from methodologically different clinical studies (double-blind, placebo-controlled study versus open-label extensions). The analysis is limited further by the uneven distribution of patients across the range of baseline NIS-LL values, with the majority of patients having a baseline NIS-LL ≤ 20. Furthermore, only neurologic functioning based on the NIS-LL and NIS-LL muscle weakness subscale were assessed in this post-hoc analysis, and other aspects of this complex illness (e.g., autonomic and/or cardiac functioning) may progress at different rates and be subject to other influences. Lastly, although the parent and extension studies were not specifically designed to assess the effect of baseline on response to treatment, including a baseline covariate to adjust for a potential baseline effect is not uncommon. The findings reported here highlight the importance of including baseline as a factor to more appropriately assess treatment effect. The results also highlight the difficulty of using a constant 2-point change from baseline (regardless of baseline severity) to define responder/non-responder status when treating ATTR-PN.

## Conclusions

This analysis of patients with Val30Met ATTR-PN demonstrates that disease progression strongly depends on baseline neurologic severity and illustrates the disease-modifying effect of tafamidis relative to placebo across a range of baseline levels of neurologic severity and treatment durations. These data also underscore the benefit of early diagnosis and treatment with tafamidis in delaying disease progression in ATTR-PN.

## Additional file

Additional file 1: **Table S1.** Independent ethics committees and institutional review boards. (DOCX 37 kb)

## Abbreviations

ATTR-PN: Transthyretin amyloid polyneuropathy
MMRM: Mixed-effects model for repeated measures
NIS–LL: Neuropathy Impairment Score–Lower Limbs
P-T: Placebo-to-tafamidis
T-T: Tafamidis-to-tafamidis
TTR: Transthyretin
Val30Met: Valine substituted for methionine at position 30
