# Supplementary material for: Influence of baseline neurologic severity on disease progression and the associated disease-modifying effects of tafamidis in patients with transthyretin amyloid polyneuropathy
Source: Orphanet J Rare Dis. 2018 Dec 17;13:225. doi: 10.1186/s13023-018-0947-7 (PMC6296038; doi:10.1186/s13023-018-0947-7)
Supplement: Supplementary file 1 — Table S1. Independent ethics committees and institutional review boards. (DOCX 37 kb) [file 13023_2018_947_MOESM1_ESM.docx]

Electronic Supplementary Material

Additional file 1

**Table S1** Independent ethics committees and institutional review boards

| Name | Location |
| --- | --- |
| Comissão de Ética para a Investigação  Clinica (CEIC) | Lisboa, Portugal |
| Hospital Geral de Santo António | Porto, Portugal |
| Hospital de Santa Maria | Lisboa, Portugal |
| CPP - Ile-de-France VI, Hôpital de la Pitié-Salpêtrière | Paris, France |
| Kings College Hospital Research Ethics Committee | London, United Kingdom |
| Regional Ethical Review Board in Umeå Campus | Umeå, Sweden |
| Comité Independiente de Ética para Ensayos en Farmacología Clínica | Buenos Aires, Argentina |
| FLENI Comité de Ética Investigaciónes Biomédicas, (CEIB) | Buenos Aires, Argentina |
| Comitê de Ética em Pesquisa do Hospital Universitário Clementino Fraga Filho (UFRJ) | Rio de Janeiro, Brazil |
| Comissaõ Nacional de Ética em Pesquisa (CONEP) | Brasilia, Brazil |
| Brigham and Women's Hospital IRB | Boston, Massachusetts, United States |
| Agencia de Ensayos Clinicos - Servicio de Farmacia | Barcelona, Spain |
| Ethik-Kommission der Aerztekammer Westfalen-Lippe  und der Medizinischen Fakultaet der Westfaelischen Wilhelms-Universitaet Muenster | Münster, Germany |
| Comitato di Bioetica della Fondazione IRCCS Policlinico S. Matteo di Pavia | Pavia, Italy |
| Johns Hopkins Medicine IRB, Office of Human Subjects Research | Baltimore, Maryland, United States |
